# Frequency, amplitude, and phase measurements in contact resonance atomic force microscopies

**DOI:** 10.3762/bjnano.5.30

**Published:** 2014-03-12

**Authors:** Gheorghe Stan, Santiago D Solares

**Affiliations:** 1 Material Measurement Laboratory, National Institute of Standards and Technology, Gaithersburg, MD 20878, USA; 2 Department of Mechanical Engineering, University of Maryland, College Park, MD 20742, USA

**Keywords:** contact-resonance AFM, dynamic AFM, frequency modulation, phase-locked loop, viscoelasticity

## Abstract

The resonance frequency, amplitude, and phase response of the first two eigenmodes of two contact-resonance atomic force microscopy (CR-AFM) configurations, which differ in the method used to excite the system (cantilever base vs sample excitation), are analyzed in this work. Similarities and differences in the observables of the cantilever dynamics, as well as the different effect of the tip–sample contact properties on those observables in each configuration are discussed. Finally, the expected accuracy of CR-AFM using phase-locked loop detection is investigated and quantification of the typical errors incurred during measurements is provided.

## Introduction

A number of atomic force microscopy (AFM) variants have emerged since the introduction of the original technique in 1986 [1]. Besides topographical acquisition and spectroscopy, an important application nowadays is the measurement of conservative and dissipative interactions across nanoscale surfaces, which is highly relevant for viscoelastic materials such as polymers and biological samples. These measurements can be carried out through a combination of contact and dynamic AFM modes. Within the force modulation method [2], the tip and the sample are brought into contact at a prescribed tip–sample force setpoint (cantilever deflection setpoint, as in contact mode imaging) and the sample is excited with a sinusoidal oscillation in the vertical direction (atomic force acoustic microscopy (AFAM) configuration [3]), such that the tip oscillation amplitude and its phase with respect to the excitation can be measured and converted into a loss and storage modulus. In contact resonance AFM (CR-AFM) [3–9] a similar setup is used, supplying the sinusoidal excitation either at the base of the cantilever (in the so-called ultrasonic atomic force microscopy (UAFM) configuration [4]) or to the sample stage (in the AFAM configuration [3]). In both cases, the effective resonance frequency, amplitude, and phase of various eigenmodes of the cantilever–tip system are generally measured through excitation frequency “sweeps” for quantitative determination of the same elastic and viscous responses of the material. More recently, other methods have been introduced to more rapidly infer the frequency response (amplitude vs frequency curves) of the tip–sample contact. In the band excitation (BE) method, a time-dependent signal containing a band of frequencies around the desired resonance is applied at each pixel of the scan, such that the frequency response at that location can be rapidly obtained through a Fourier transform of the cantilever tip response and a fit to a Lorentzian curve [10–11]. This calculation allows mapping of the resonance frequency and quality factor across the sample, from which viscoelastic properties can also be inferred. In contrast, in the dual-amplitude resonance tracking (DART) method, the frequency response curve is rapidly inferred from the phase and amplitude response at two frequencies around the resonance frequency during a real-time scan [12].

Intermittent-contact methods have also been used to characterize conservative and dissipative tip–sample interactions simultaneously with topographical acquisition. This was originally performed using the tapping-mode (amplitude modulation) technique [13], within which variations in the phase contrast can be directly related to changes in energy dissipation [14–15]. Conservative and dissipative interactions are generally expressed in terms of the virial (*V*_ts_) and the dissipated power (*P*_ts_), respectively [15–20]. In the last ten years, intermittent-contact measurements have been enhanced through multifrequency excitation methods [21–27]. In multifrequency AFM, the fundamental cantilever eigenmode is typically controlled in conventional AM- or FM-AFM mode for topographical measurement, while one or more higher eigenmodes are driven simultaneously in order to also map compositional (viscoelastic) contrast. Since the higher eigenmodes are not directly affected by the topographical acquisition controls, they can be tuned independently to map *V*_ts_ and *P*_ts_ with high sensitivity. However, with the exception of small-amplitude FM-AFM [28–29] in which the tip–sample force gradient can be measured directly, the mapping of *V*_ts_ and *P*_ts_ in intermittent-contact imaging generally only provides a qualitative map of surface viscoelasticity.

In this work the focus is on the CR-AFM technique. Specifically, we analyzed the response variables for the two configurations currently in use (UAFM and AFAM), and restricted our analysis to the first two cantilever eigenmodes. Similarities and notable differences were observed in the signals and calculated variables (frequency, amplitude and phase) for the two cases, which require careful analysis for proper experimental setup and interpretation. As an example, we analyzed the errors introduced during resonance frequency tracking through the use of a phase-locked loop (PLL), which leads to different results in both configurations. This is a highly relevant practical consideration, since PLL techniques offer versatility and speed of characterization when they can be implemented accurately.

## Results and Discussion

### Equation of motion for a cantilever beam in UAFM and AFAM configurations

**Figure 1:**
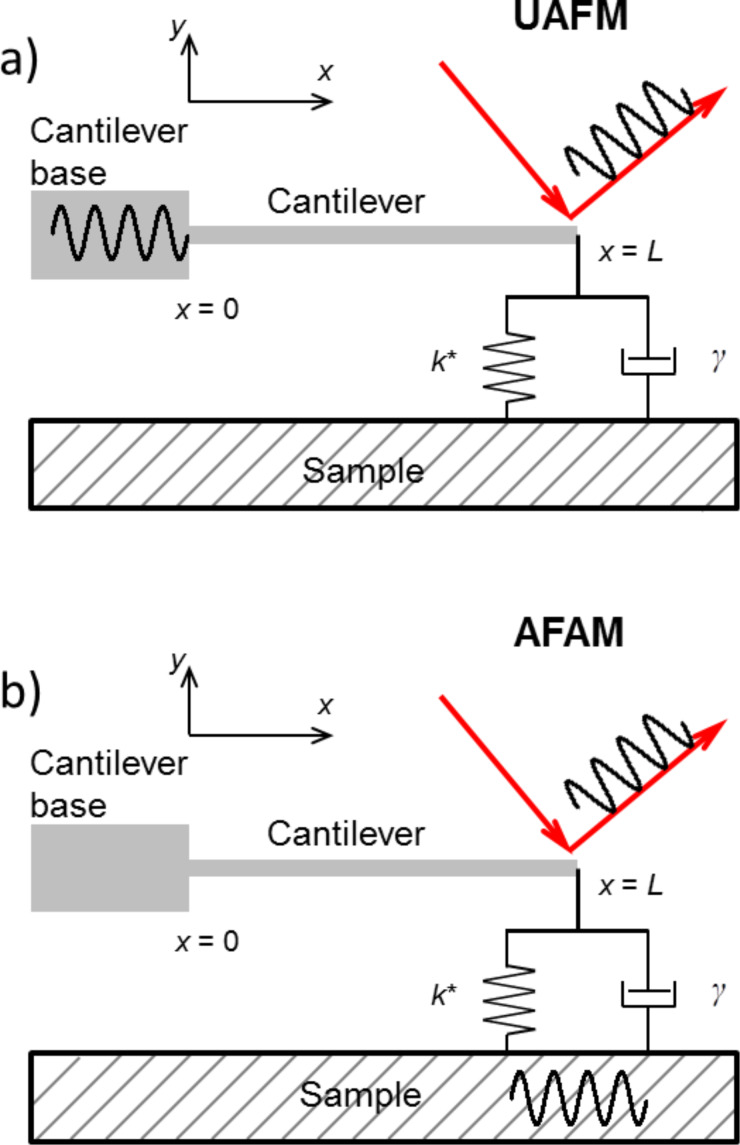
a) UAFM configuration with a mechanical vibration applied to the base of the cantilever and signal detection at the end of the cantilever. b) AFAM configuration with a mechanical vibration applied to the sample and signal detection at the end of the cantilever.

In this work two CR-AFM configurations will be analyzed: UAFM [4], with the cantilever vibrated from its base (Figure 1a), and AFAM [3], with the sample vibrated from underneath (Figure 1b). In both configurations the vibration is in the form of a mechanical oscillation of variable frequency and the detection is performed at the end of the cantilever where the tip is located. The dynamics of the cantilever–tip–sample system in each of these configurations was discussed by Rabe in [30]. We limit ourselves to briefly reviewing the equations necessary for our analysis. For simplicity, the vertical tip–sample coupling was modelled as a spring in parallel with a dashpot (Kelvin–Voigt model) and no lateral contact coupling was considered; vertical and lateral refer here to the normal and parallel directions to the sample surface, respectively.

The Euler–Bernoulli equation of motion for damped flexural vibrations of a cantilever beam in air is

[1]
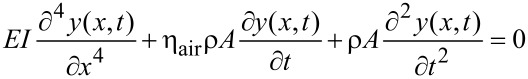


where the cantilever is described by its Young’s modulus *E*, second moment of area of its cross section *I*, mass density ρ, and cross-sectional area *A*, and η_air_ characterizes the damping of the oscillations in air. The general solution of Equation 1 is in the form of *y*(*x*,*t*) = *y*(*x*)*e^i^*^ω^*^t^*, with

[2]
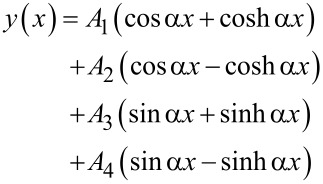


with *A*_1_, *A*_2_, *A*_3_, and *A*_4_ constants and α the complex wave number of a flexural oscillation, 
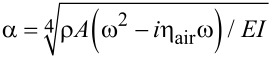
.

For the UAFM and AFAM configurations shown in Figure 1, the following boundary conditions are imposed to the general solution:

[3]
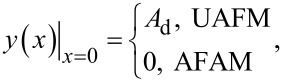


[4]
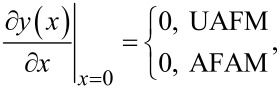


[5]
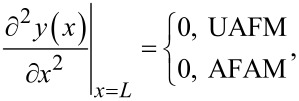


and

[6]
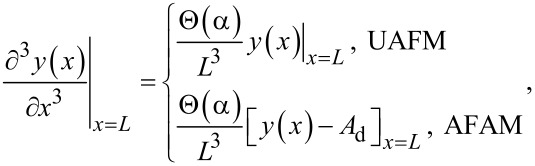


where *L* is the length of the cantilever, *A*_d_ the driven amplitude, and Θ(α) is given by

[7]
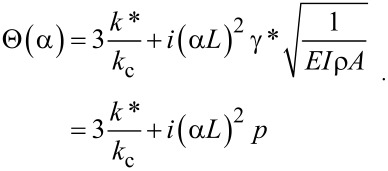


Here *k*_c_ = 3*EI*/*L*^3^ is the cantilever spring constant, k* the contact stiffness, γ* the contact damping constant, and 
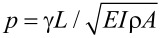
 the dimensionless contact damping constant. With the above specified boundary conditions the solution further simplifies to

[8]
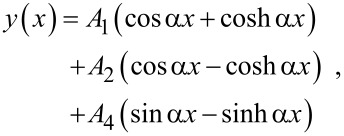


with the following constants for the two configurations:

[9]
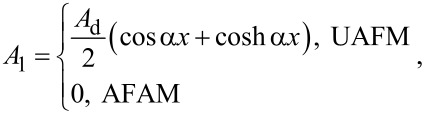


[10]
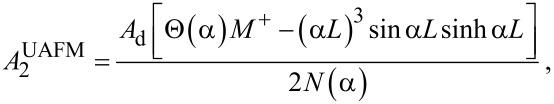


[11]
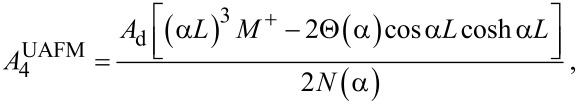


[12]
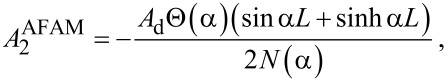


and

[13]
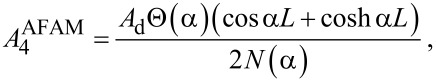


with *M*^±^ = sin α*L* cosh α*L* ± sinh α*L* cos α*L*, *N*(α) = (α*L*)^3^ (1 + cos α*L* cosh α*L*) + Θ(α)*M*^−^, and Θ(α) given by Equation 7. In particular, the deflection of the end of the cantilever reduces to

[14]
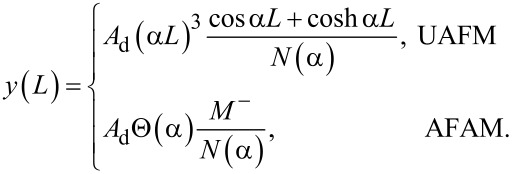


The magnitude of the deflection and phase are given by:

[15]
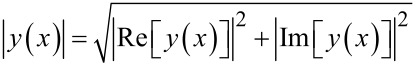


and

[16]
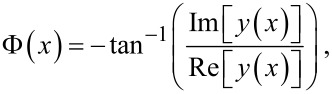


respectively.

**Figure 2:**
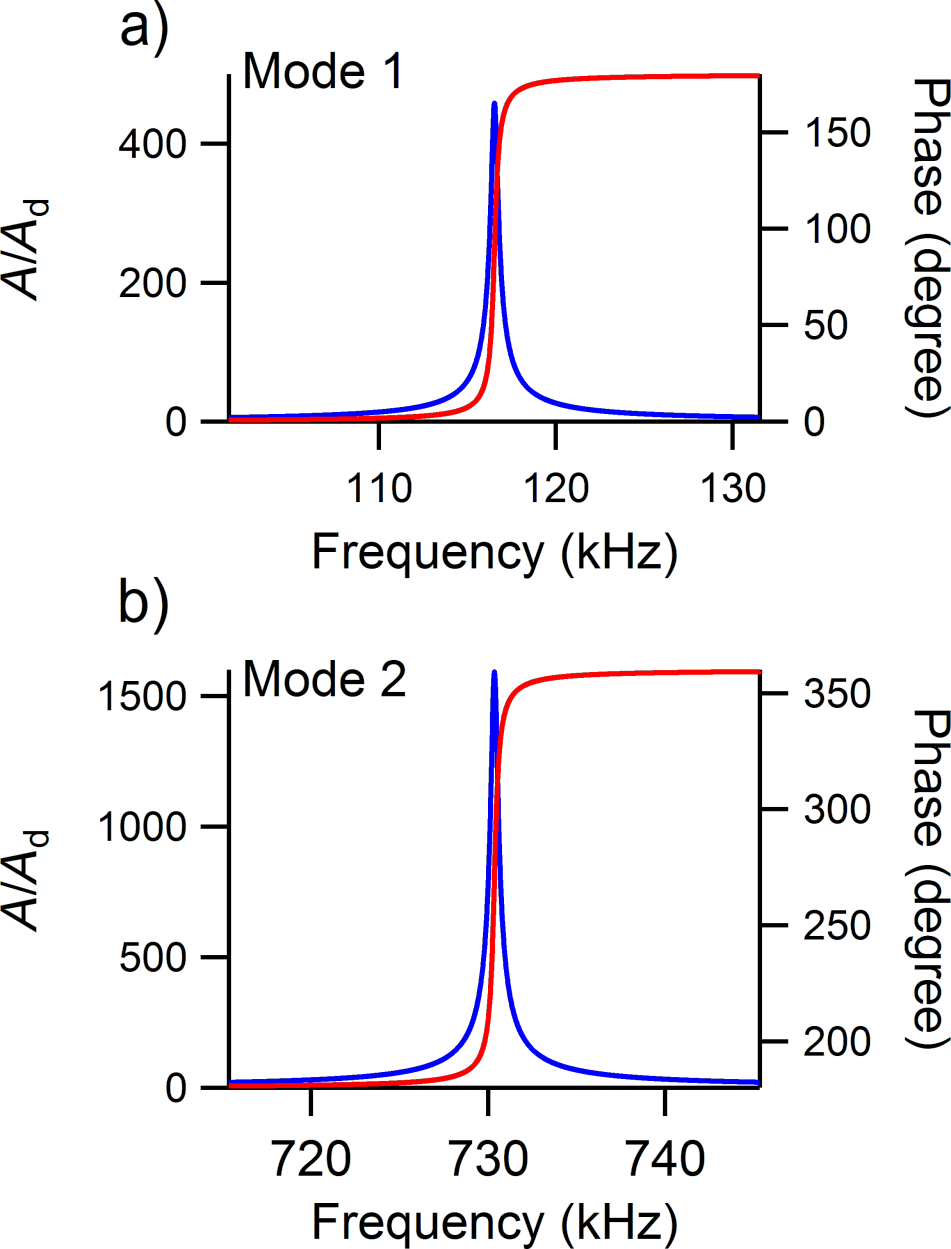
Amplitude ratio and phase of the a) first and b) second free eigenmodes of a cantilever vibrated in the UAFM configuration, measured at the tip.

We illustrate our analysis with a rectangular Si cantilever of length *L* = 225.03 µm, width *w* = 30.00 µm, and thickness *T* = 4.89 µm. With mass density ρ_Si_ = 2329.00 kg/m^3^ and Young’s modulus *E*_Si_ = 130.00 GPa, the cantilever’s spring constant was calculated as *k*_c_ = 10.00 N/m. Using these parameters and considering η_air_ = 2.50 s^−1^ in Equation 1, the first two eigenmodes are characterized by the dynamic parameters given in Table 1. The frequency dependences of the amplitude ratio and phase around resonance are shown in Figure 2 for the first two free eigenmodes of the cantilever. For calculations of the free-eigenmodes, the cantilever was vibrated in the UAFM configuration. In the following analysis we will characterize the contact damping by the dimensionless contact damping constant *p* rather than the actual contact damping constant γ*. The discussion is focused on the dynamics of the cantilever in the two CR-AFM configurations only and further consideration of various contact geometries would be required to convert the measured dynamic parameters into the elastic and viscous properties of the materials and structures probed [8–9,31–33].

**Table 1:** Cantilever parameters.

	Mode 1	Mode 2

Resonance frequency (kHz)	116.54	730.37
Amplitude ratio^a^	458.69	1593.10
Phase (degree)	90.05	270.01
Quality factor *Q*	292.90	1835.64

^a^The amplitude ratio refers to the amplitude at resonance, *A*, normalized to the driven amplitude, *A*_d_.

### Amplitude and phase along the cantilever

**Figure 3:**
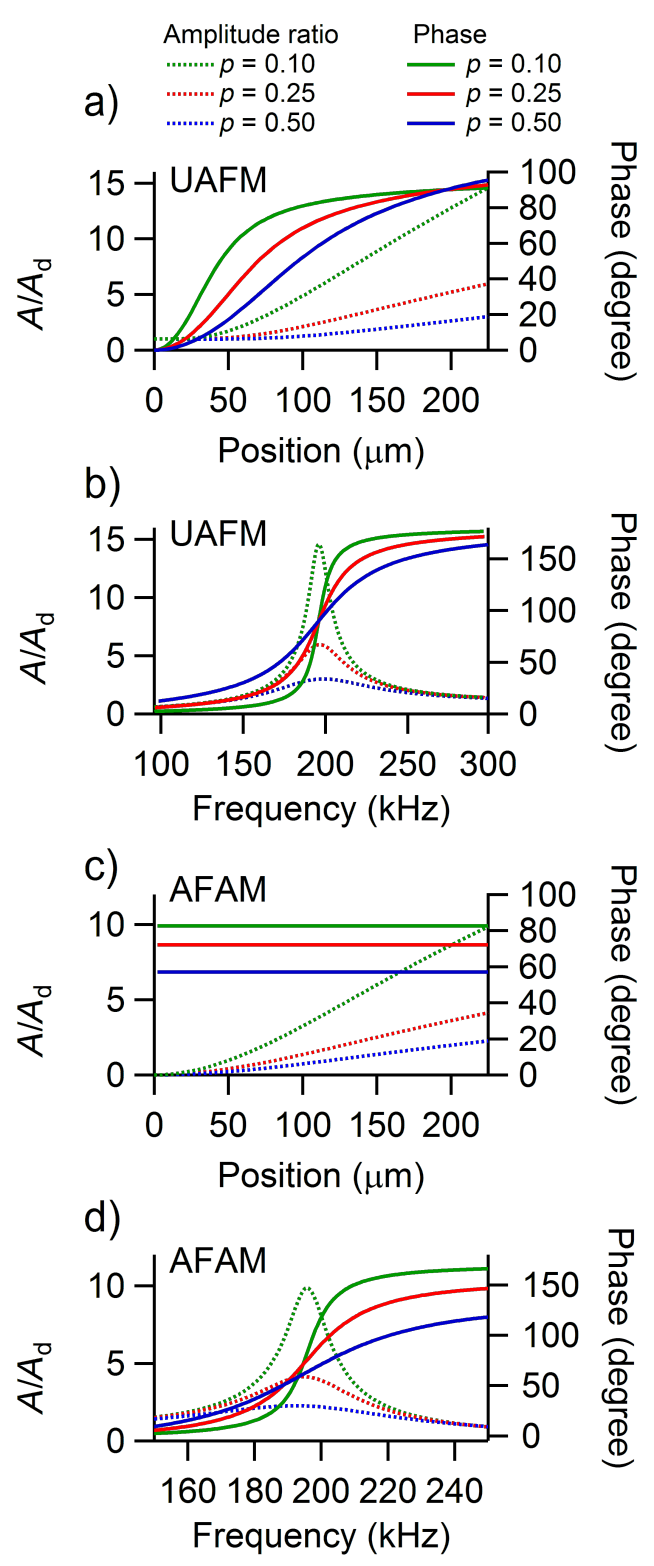
Amplitude ratio and phase of the first eigenmode along the cantilever in a) the UAFM and c) AFAM configurations, respectively. Frequency dependence of the amplitude ratio and phase of the first eigenmode at the end of the cantilever in b) the UAFM and d) AFAM configurations, respectively.

In Figure 3 are shown the amplitude ratio and phase of the first eigenmode along the cantilever for the UAFM (Figure 3a) and AFAM (Figure 3c) configurations for the same contact stiffness, k* = 20 N/m, and three different contact damping values: mild (*p* = 0.10), medium (*p* = 0.25), and strong (*p* = 0.50) contact damping. In both configurations, the calculated displacement along the cantilever shows the deformed shape of the first eigenmode with a node at the base of the cantilever (*x* = 0) and an antinode at the end of the cantilever (*x* = *L*), with smaller and smaller displacement values as the contact damping increases. In contrast to the displacements, the phase response is quite different in magnitude and shape. Thus, in the UAFM configuration, the phase of the first eigenmode (refer to Figure 3a) goes from 0 at the base of the cantilever to around 90 degrees at the end of the cantilever. The resonance state at the end of the cantilever for the UAFM configuration is detailed in Figure 3b in terms of amplitude and phase. From this, little change in the phase can be observed for the range of considered contact damping, from 91.1 degrees for *p* = 0.10 to 95.5 degrees for *p* = 0.50. Interestingly, as can be seen in Figure 3a, the phase is about 90 degrees at 87% of the length of cantilever, independent of the contact damping values. The key observation here is that the phase at the end of the cantilever in the UAFM configuration varies by a few degrees around 90 degrees depending on the magnitude of the contact damping. However, a completely different response in phase is shown in Figure 3c and 3d for the AFAM configuration. First, the phase of the first AFAM eigenmode is essentially constant (very small variation) along the cantilever. Second, its magnitude changes significantly with the considered contact damping. It decreases from essentially 90 degrees when no contact damping is present to 82.6 for *p* = 0.10, to 72.1 degrees for *p* = 0.25, and to 57.1 degrees for *p* = 0.50.

**Figure 4:**
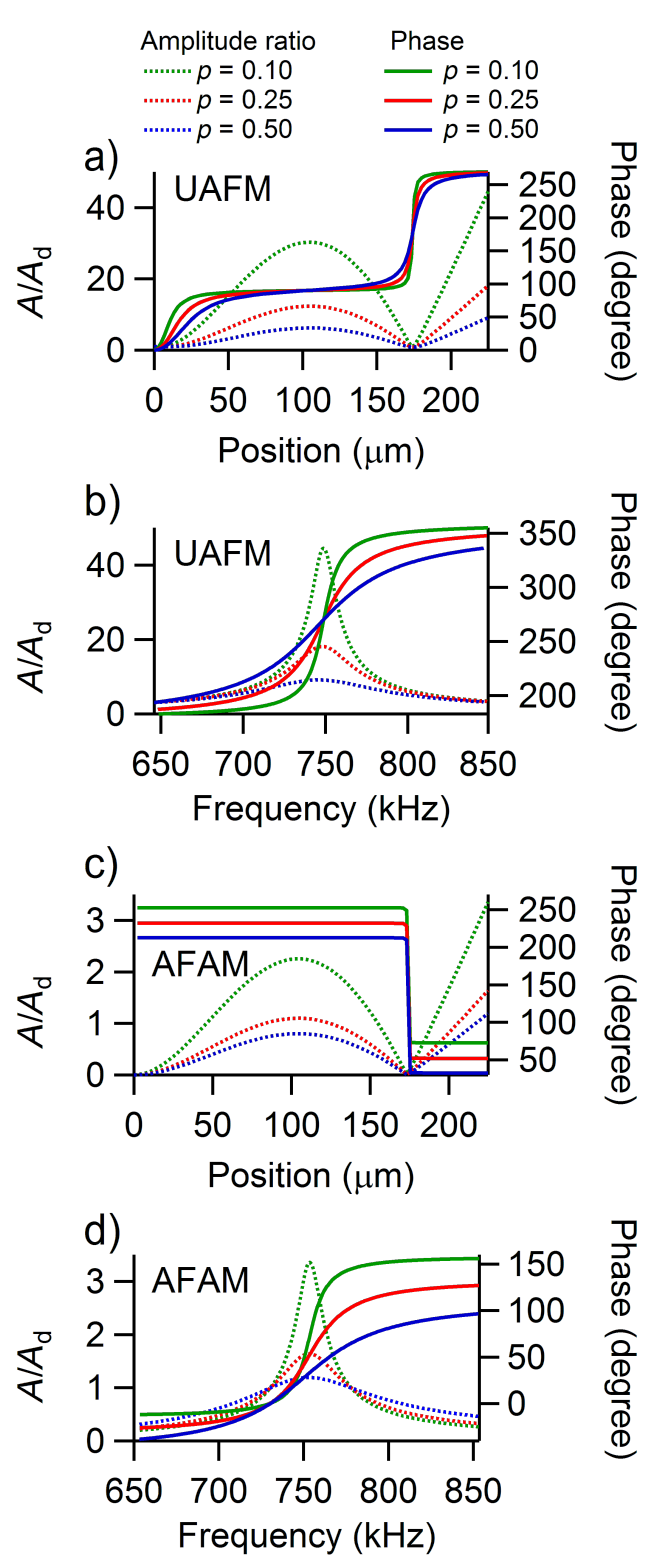
Amplitude ratio and phase of the second eigenmode along the cantilever in a) the UAFM and c) AFAM configurations, respectively. Frequency dependence of the amplitude ratio and phase of the second eigenmode at the end of the cantilever in b) the UAFM and d) AFAM configurations, respectively.

An analogous analysis can be carried out for the amplitude and phase of the second eigenmode shown in Figure 4. The shape of the second eigenmode of the cantilever exhibits two nodes (at the base of the cantilever and at 77% of the length of the cantilever) and two antinodes (at 46% of the length of the cantilever and at the end of the cantilever). Both the UAFM and AFAM configurations impose the same shape for the second eigenmode but the amplitude is about one order of magnitude larger in UAFM than in AFAM. As in the case of the first eigenmode discussed above, the phase of the second eigenmode differs substantially between the two configurations. In the UAFM configuration, the phase is 0 at the cantilever base, shows a 90 degrees plateau around the first antinode, goes through 180 degrees at the second node, and shows another plateau of 270 degrees at the end of the cantilever; 270 degrees is equivalent here to a resonance at −90 degrees. As observed in Figure 4a at the end of the cantilever and also in Figure 4b from the frequency dependences around the resonance, the phase of the second eigenmode at the end of the cantilever experiences small variations as a function of contact damping: 269.1 degrees for *p* = 0.10 to 265.4 degrees for *p* = 0.50. In the AFAM configuration, the phase resembles the shape of a two-step function with a sharp transition at the second node. At the end of the cantilever, the phase of the second AFAM eigenmode shown in Figure 4c and 4d varies substantially with the contact damping considered: From 72.6 degrees for *p* = 0.10, to 52.0 degrees for *p* = 0.25, and to 32.6 degrees for *p* = 0.50.

From the above discussion of the amplitude and phase of the first and second eigenmodes of the cantilever, we can conclude that for a given contact stiffness, the amplitude changes significantly with the contact damping and this change is qualitatively and quantitatively similar in UAFM and AFAM. However, the phases of the two configurations differ significantly from each other. In the UAFM configuration the phase experiences small variations as a function of contact damping, with values around 90 degrees (first eigenmode) or −90 degrees (second eigenmode). On the other hand, in the AFAM configuration, the phase is very sensitive to changes in contact damping and exhibits large variations. This analysis indicates that both the UAFM and AFAM amplitudes but only the AFAM phase are good measurable quantities for determining the contact damping of the tip–sample coupling. On the other hand, the UAFM phase is quite insensitive to the contact damping and it would not be a good measurement for it. However, as discussed later, the invariance of the UAFM phase to contact damping can be used to track the resonance state by phase-control techniques (i.e., PLLs) [34–35].

### Contact resonance frequency, amplitude, and phase

To retrieve the contact stiffness and contact damping responses of a material, measurements are made in terms of resonance frequency, amplitude, and phase in any of the CR-AFM configurations. In the following we will analyze these various signals at the end of the cantilever as a function of contact stiffness and contact damping in UAFM and AFAM configurations and examine the differences between these two configurations.

**Figure 5:**
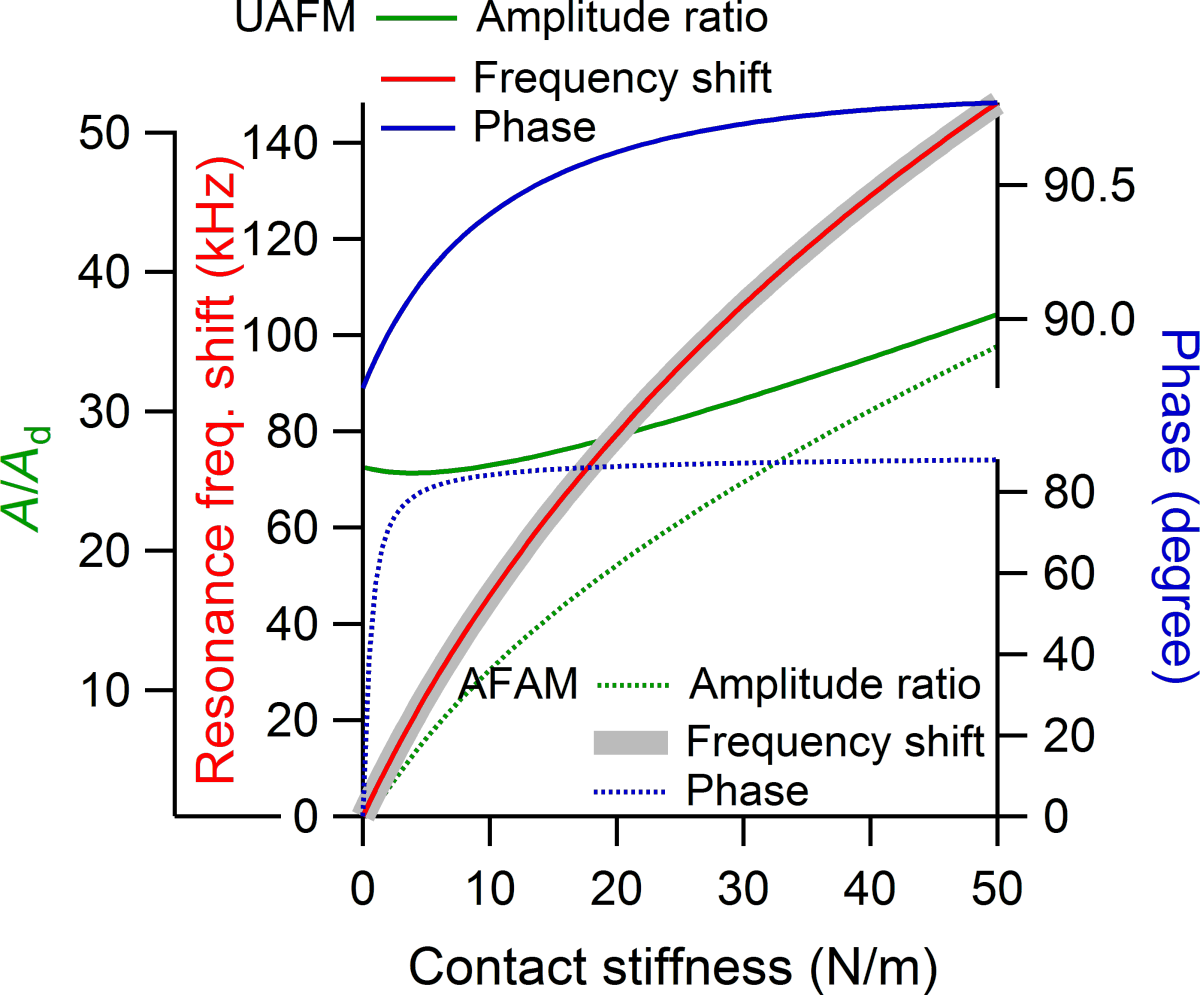
Amplitude ratio, frequency shift, and phase of the first eigenmode versus contact stiffness in UAFM and AFAM configurations when a small contact damping of *p* = 0.05 was considered.

**Figure 6:**
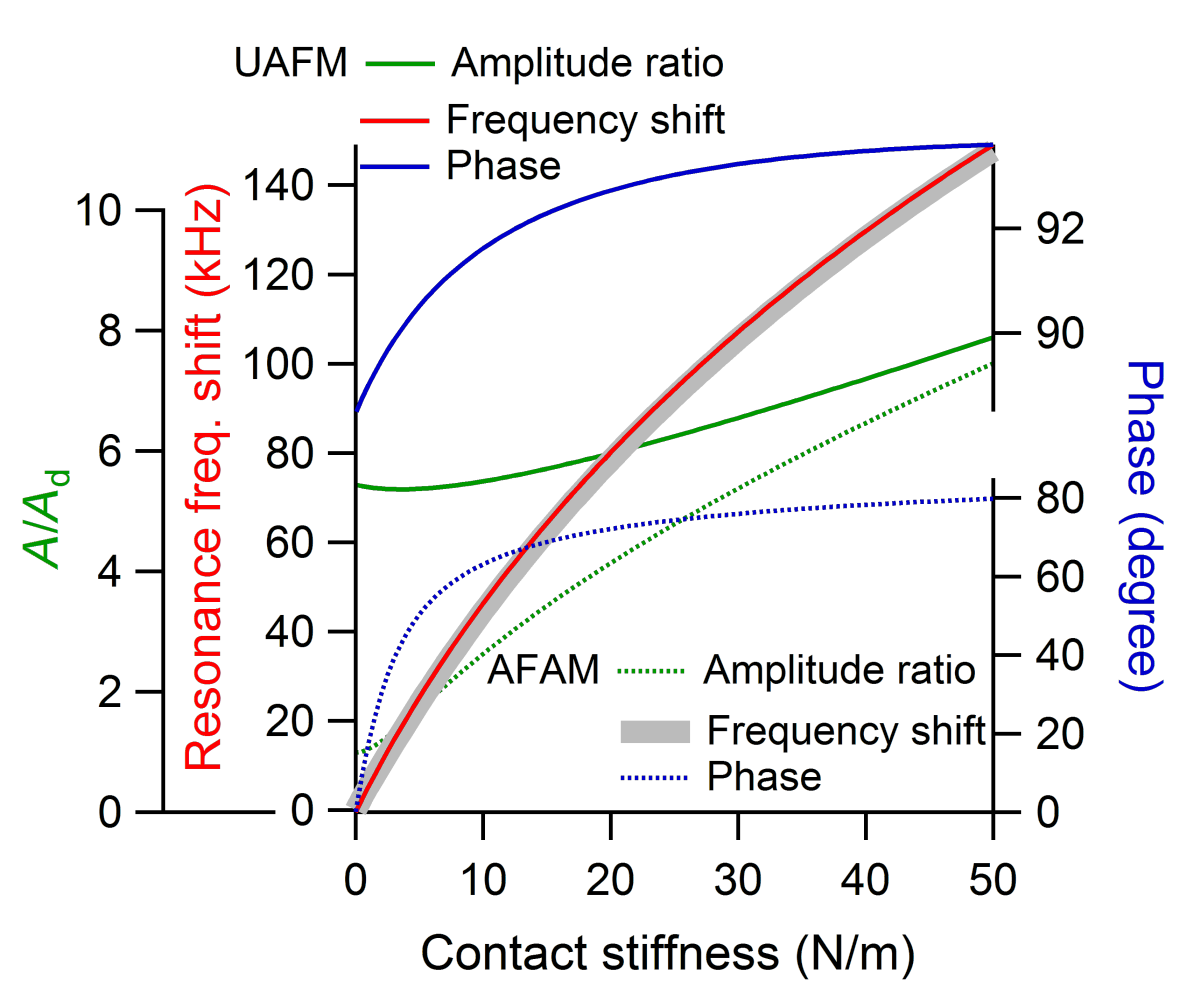
Amplitude ratio, frequency shift, and phase of the first eigenmode versus contact stiffness in UAFM and AFAM configurations when a medium contact damping of *p* = 0.25 was considered.

The amplitude ratio, resonance frequency, and phase of the first eigenmode are shown as a function of the contact stiffness in Figure 5 for a small *p* = 0.05 contact damping and in Figure 6 for a medium *p* = 0.25 contact damping, respectively. All the cantilever parameters were taken to be the same as above, with *k*_c_ = 10.00 N/m. As can be seen in Figure 5 and Figure 6, for each of the contact damping values considered, there is no significant difference between the UAFM and AFAM resonance frequencies (red and grey continuous lines) over the investigated contact stiffness range. This shows that in terms of contact stiffness measurements based on the shift in the resonance frequency the UAFM and AFAM configurations provide the same result. The differences between the two configurations are notable in terms of amplitude and phase. In the UAFM configuration, the amplitude (green continuous line in Figure 5 and Figure 6) slowly increases with the increase in contact stiffness. For the two contact damping values considered in Figure 5 and Figure 6, the overall increase in UAFM amplitude was about 40% between the initial value at k* = 0 N/m and end value at k* = 50 N/m. A more abrupt increase can be observed for the AFAM amplitude (green dotted lines in Figure 5 and Figure 6). In the AFAM configuration the amplitude is zero at k* = 0 N/m when the tip and the sample are basically uncoupled. In practice, however, small oscillations are induced in the cantilever when it is brought close to but still not in contact with the vibrated sample. So, in this case of very small contact stiffnesses, the theoretical AFAM configuration might not be reproduced in experiments. It is interesting to observe that the UAFM and AFAM amplitudes become comparable towards large contact stiffness couplings in both cases of small and medium contact damping. The phase variation as a function of contact stiffness is similar to the amplitude variation in each configuration. Thus, over the considered contact stiffness range, the UAFM phase (blue continuous line in Figure 5 and Figure 6) changes within one degree from its free value (90 degrees) in the case of a small *p* = 0.05 contact damping and within 4 degrees in the case of a medium *p* = 0.25 contact damping. However, a much larger variation is experienced by the AFAM phase (dotted blue line in Figure 5 and Figure 6) with the increase in the contact stiffness. From essentially zero degrees, in the absence of tip–sample coupling, the AFAM phase increases sharply in the range of small contact stiffnesses and has an asymptotical increase for contact stiffnesses comparable or larger than the cantilever stiffness. These asymptotic values of the AFAM phase however depend strongly on the actual contact damping. For the examples shown in Figure 5 and Figure 6, the AFAM phase approaches 87 degrees for a contact stiffness of *p* = 0.05 and 80 degrees for a contact stiffness of *p* = 0.25. This reiterates the above observation that the AFAM phase is sensitive to contact damping and could be used as a measure of the tip–sample contact damping.

**Figure 7:**
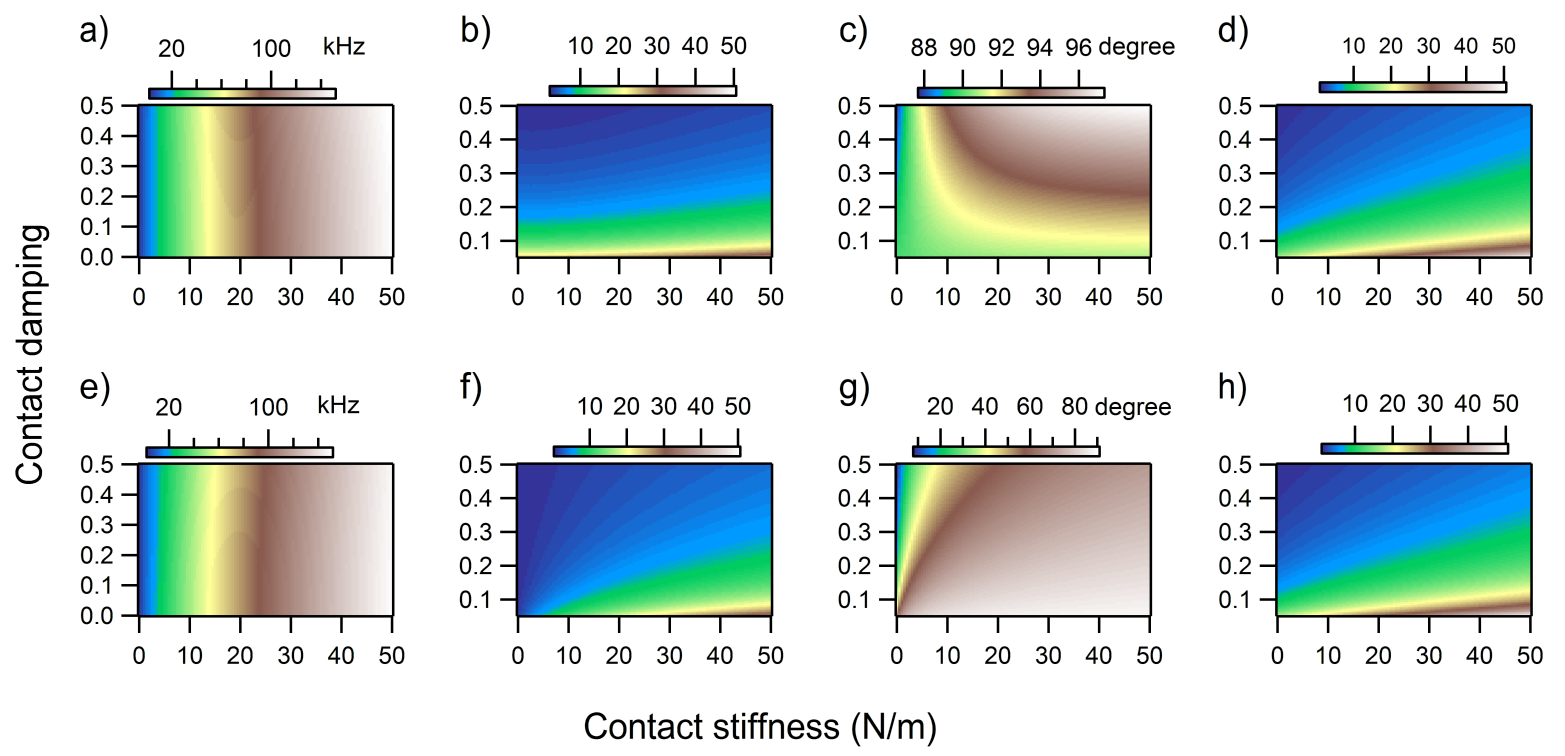
a) Frequency shift, b) normalized amplitude, c) phase, and d) quality factor *Q* of the first eigenmode in the UAFM configuration as a function of contact stiffness and contact damping. e) Frequency shift, f) normalized amplitude, g) phase, and h) quality factor *Q* of the first eigenmode in the AFAM configuration as a function of contact stiffness and contact damping.

**Figure 8:**
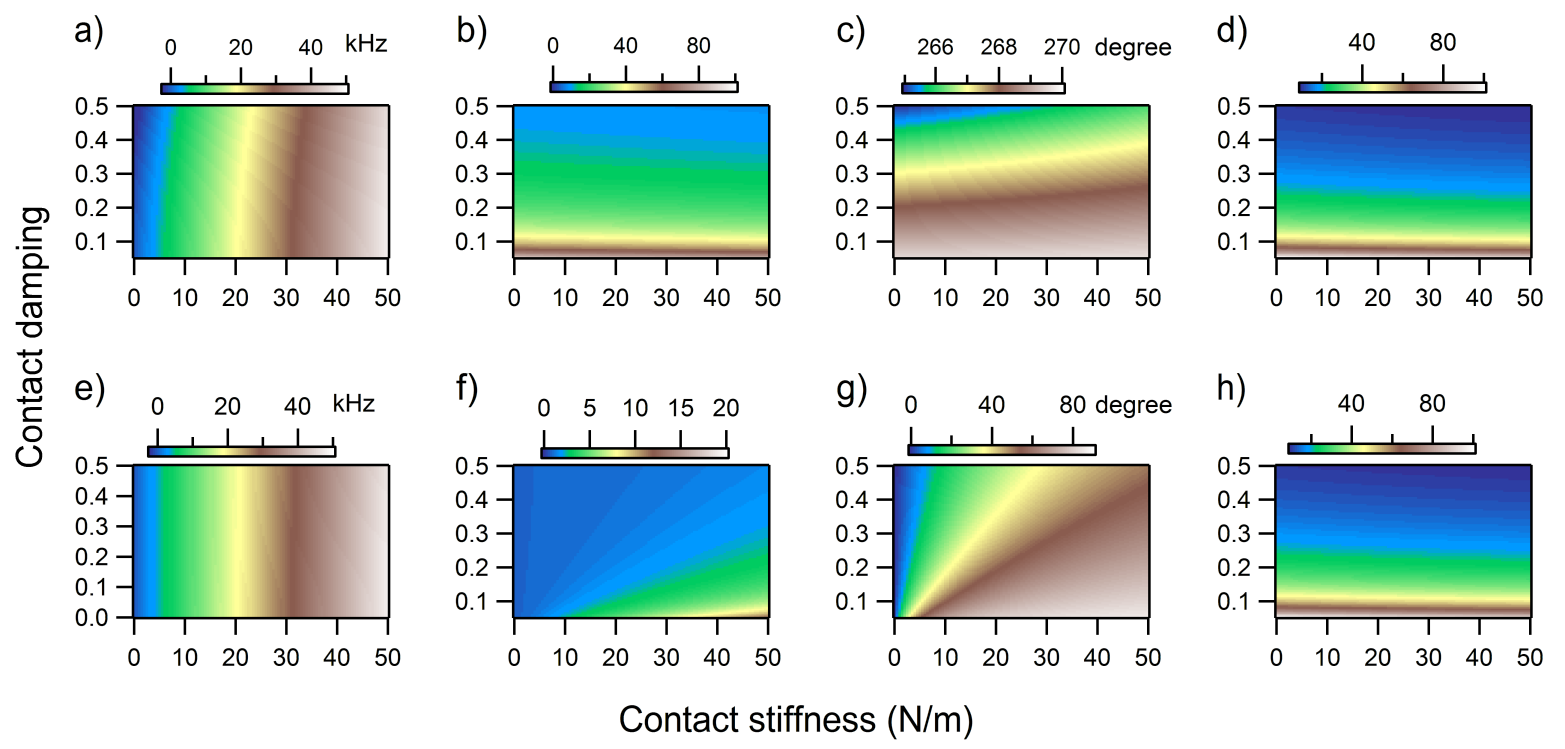
a) Frequency shift, b) normalized amplitude, c) phase, and d) quality factor *Q* of the second eigenmode in the UAFM configuration as a function of contact stiffness and contact damping. e) Frequency shift, f) normalized amplitude, g) phase, and h) quality factor *Q* of the second eigenmode in the AFAM configuration as a function of contact stiffness and contact damping.

The variations of the contact resonance frequency, amplitude, and phase as a function of both contact stiffness and contact damping were fully analyzed in the maps shown in Figure 7 for the first eigenmode and in Figure 8 for the second eigenmode of UAFM and AFAM, respectively. In terms of contact resonance frequency, large shifts were observed over the range of considered contact stiffness and damping: about 130 kHz for the first eigenmode (Figure 7a and 7e) and about 50 kHz for the second eigenmode (Figure 8a and 8e). As can be seen, the frequency shifts are almost insensitive to contact damping and mainly responsive to contact stiffness variations only. On the other hand, a pronounced contact damping dependence and moderate contact stiffness dependence can be observed in the amplitude maps (Figure 7b and 7f for the first eigenmode and Figure 8b and 8f for the second eigenmode), especially for the UAFM configuration. With the exception of the small contact stiffness range, the UAFM and AFAM amplitude values are comparable for the first eigenmode (Figure 7b and 7f). In the case of the second eigenmode, the UAFM amplitudes are consistently larger than the AFAM amplitudes, exhibiting a better amplitude detection of the second UAFM eigenmode than its counterpart in the AFAM configuration. A concurrent dependence on contact stiffness and contact damping can be observed in the maps of the phase at resonance (Figure 7c and 7g for the first eigenmode and Figure 8c and 8g for the second eigenmode). The UAFM phase response to the considered contact stiffness and contact damping variations is of order of a few degrees around 90 degrees for the first eigenmode and few degrees below 270 degrees (−90 degrees) for the second eigenmode. Thus, the UAFM phase of the first eigenmode (Figure 7c) is less than 90 degrees for compliant materials with either low or high contact damping and stiff materials with low contact damping. The phase goes above 90 degrees in the less realistic case of stiff materials with high damping. An even smaller variation of only 5 degrees below the free resonance phase was observed for the second UAFM eigenmode (Figure 8c). As inferred from the above discussion, the AFAM phase, either for the first eigenmode (Figure 7g) or second eigenmode (Figure 8g) exhibits large variation as a function of contact stiffness and contact damping. Thus, the AFAM phase is around zero degrees at small contact stiffnesses and goes asymptotically towards 90 degrees as the contact stiffness increases. This asymptotic trend is progressively delayed with the increase in contact damping. An interesting behaviour is observed also in the maps of quality factor *Q* (Figure 7d and 7e for the first eigenmode and Figure 8d and 8e for the second eigenmode), calculated as the ratio of the resonance frequency to the width of the resonance peak, ω_n_/Δω. In general, the quality factor is directly associated with the damping response of the system. However, as it can be seen in Figure 7d and 7h, it depends on both the contact stiffness and contact damping. The *Q*-factor is almost independent of contact stiffness for the second UAFM and AFAM eigenmodes, in which case it can be used as a direct measurement of the tip–sample contact damping. Explicit relationships between the *Q*-factors of various contact eigenmodes and contact damping were intuitively proposed [36] and rigorously derived [37] previously for the AFAM configuration. The results shown in Figure 7h and Figure 8h are in agreement, within the common range of contact stiffness, with the *Q*-factor versus contact damping dependences shown in Figure 2 of [37] for the first two eigenmodes.

### Phase-locked loop detection

By considering their specific dependences in either UAFM or AFAM configurations, the measured contact resonance frequency, amplitude, and phase can be converted into the stiffness and damping of the tip–sample contact coupling. One way of observing the fast change in the dynamics of a cantilever used in CR-AFM point measurements or scanning is to track the resonance state by PLL detection, similar with what is used in non-contact frequency modulation AFM. In non-contact AFM, PLL tracking has been implemented in either constant-excitation frequency modulation [17–18] or constant-amplitude frequency-modulation [19–20]. In the following we will refer only to the constant-excitation PLL setup in which the driving amplitude is constant and the frequency is adjusted continuously to maintain a constant phase difference between drive and response, φ_PLL_. In the case of an AFM cantilever brought into contact from air, the PLL reference phase would be the phase of the free oscillation of the selected eigenmode. However, as we discussed above, the phase of a vibrated cantilever that is in contact with a sample, even when it is driven at the resonance, is not constant but varies in accordance with the magnitudes of the contact stiffness and contact damping. This means that in PLL detection the true resonance condition will not be retrieved. Instead one would obtain the state having the predefined PLL phase, φ_PLL_. The error introduced by the PLL in measuring the resonance frequency will then by Δ*f* = *f*_resonance_ − *f*_PLL_, where *f*_resonance_ is the dynamic resonance frequency and *f*_PLL_ is the frequency at which the phase of the detected signal is φ_PLL_.

**Figure 9:**
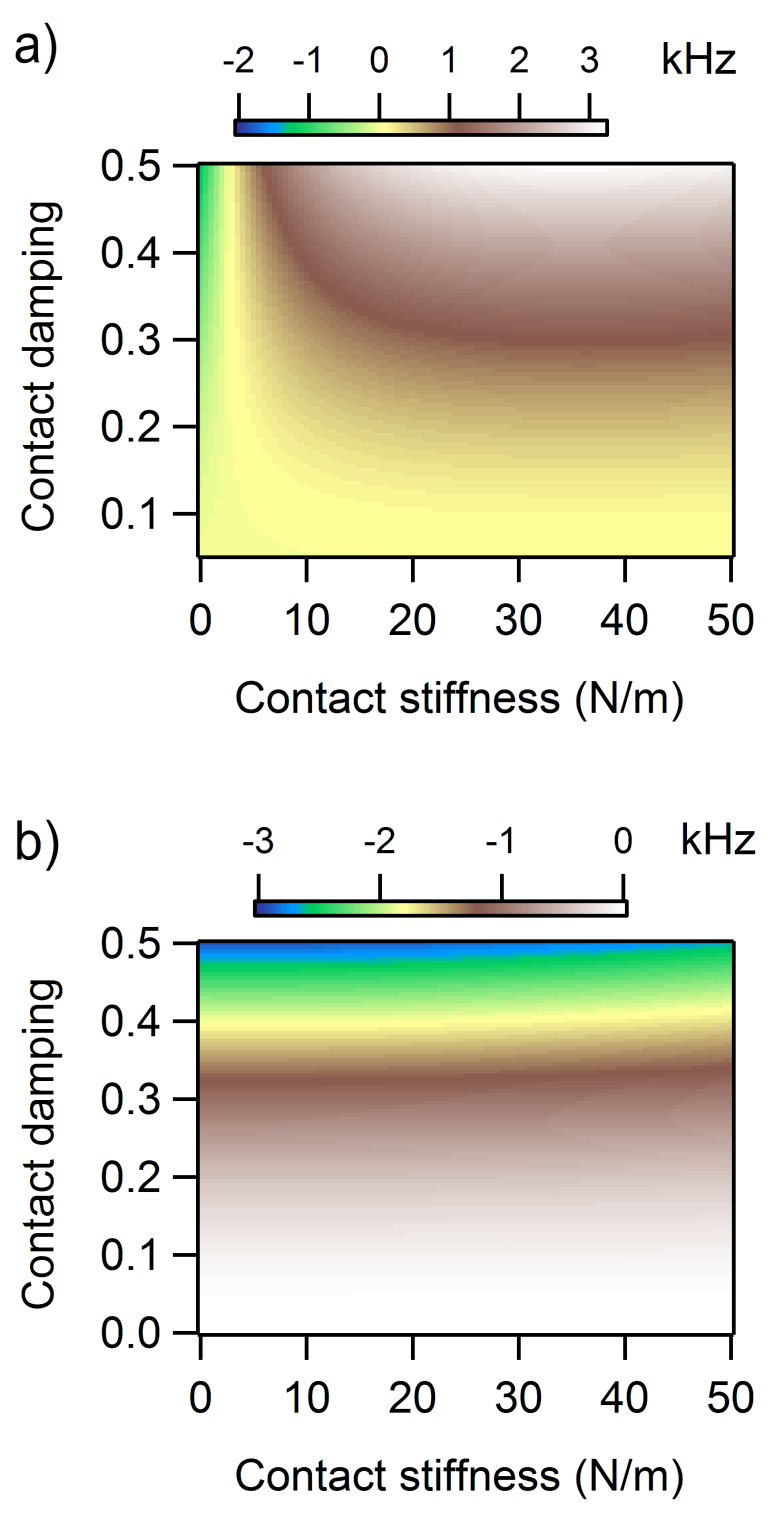
The frequency error introduced by a PLL in measuring the shift of the contact resonance frequency of (a) the first and (b) second eigenmodes in the UAFM configuration as a function of contact stiffness and contact damping. The corresponding frequency shifts over the investigated contact stiffness and contact damping ranges are shown in Figure 7a and Figure 8a for the two eigenmodes, respectively.

Based on its weak dependence on contact stiffness and contact damping, the UAFM phase can be used in a PLL detection [35,38] to maintain the cantilever–tip–sample system at the resonance and track the changes in the resonance frequency and amplitude. Figure 9 shows the errors introduced by the PLL in measuring the resonance frequency of the first and second eigenmodes when the locked phase was that of the free resonance of the respective eigenmode. As can be seen in Figure 9, the errors introduced by the PLL in determining the true resonance frequencies of the first two UAFM eigenmodes are within 1 kHz for low and medium contact damping (*p* < 0.25) over the contact stiffness range considered. In the case of very large contact damping, these errors extend to about 2 kHz or 3 kHz for some particular values of contact stiffness. Considering that these errors are for shifts of about 150 kHz for the resonance frequency of the eigenmode (refer to Figure 7a) and 50 kHz for the resonance frequency of the second mode (refer to Figure 8a), respectively, they result in negligible errors in the conversion of measured contact resonance frequencies into material elastic moduli.

A particular situation arises in the case of using PLL detection in the AFAM configuration. As was discussed above, large variations are experienced by the AFAM phase from out of contact to contact states. In the AFAM configuration the phase was found to be very sensitive to the stiffness and damping of the tip–sample contact. This phase sensitivity could be used directly for contact damping measurements [8] but would make impractical the PLL detection of the contact resonance of an AFAM eigenmode with respect to its free resonance. However, a moderate variation is experienced by the AFAM phase for contact stiffness comparable or greater than the stiffness of the cantilever (e.g., contact stiffnesses about or greater than 10 N/m in the examples considered in Figure 5 and Figure 6). It is therefore possible to perform PLL tracking even in the AFAM configuration by choosing a reference contact resonance state with respect to which moderate phase variations are experienced during contact measurements or scanning. This type of measurement has been performed also in the UAFM configuration of CR-AFM on Cu-low-*k* dielectric materials, with the PLL locked on the phase of a contact resonance state, after the tip was brought into contact at the desired applied force [35]. From a practical point of view, it is worth mentioning here that in the case of UAFM, the detection is very sensitive to the transfer function of the cantilever used and in some cases, depending on the cantilever used and tip–sample couplings, spurious resonances can mask or distort the real tip–sample coupling resonances [39–40]. On the other hand, in AFAM configuration, the frequency spectra are heavily overwritten by the transfer function of the excitation actuator (underneath the sample), which can provide cleaner spectra at the expense of a more aggressive tip–sample coupling.

## Conclusion

The resonance frequency, amplitude, and phase of the first two eigenmodes of two contact resonance AFM (CR-AFM) configurations, namely a setup with sample stage excitation (AFAM) and one with cantilever base excitation (UAFM), were analyzed in detail. This allowed observing similarities and differences among the dynamic parameters of each of the CR-AFM configurations as a function of the mechanical coupling on different materials. Thus, while the contact resonance frequency is mostly sensitive to contact stiffness and less sensitive to contact damping, the resonance amplitude and phase exhibit a concurrent dependence on both contact stiffness and contact damping. Also, it was found that the two CR-AFM configurations differ greatly through their phase response. Thus, while the UAFM phase shows a reduced variation over a large range of material parameters, the AFAM phase is very sensitive to both contact stiffness and contact damping. These results suggest that, from an experimental point of few, UAFM would be the preferred CR-AFM configuration in phase-control detection applications. However, with appropriate use of their specific frequency dependences, both amplitude and phase are theoretically available for elastic modulus and dissipation measurements in both UAFM and AFAM configurations.
